# High frequency of type I interferon auto‐antibodies in a group of middle‐aged HIV‐infected patients: A cross‐sectional exploratory study

**DOI:** 10.1002/iid3.1056

**Published:** 2023-11-22

**Authors:** Luisa Imberti, Paola Magro, Alessandra Sottini, Virginia Quaresima, Francesco Castelli, Eugenia Quiros‐Roldan

**Affiliations:** ^1^ Section of Microbiology University of Brescia Brescia Italy; ^2^ Department of Infectious and Tropical Diseases University of Brescia Brescia Italy; ^3^ Clinical Chemistry Laboratory ASST Spedali Civili of Brescia Brescia Italy

**Keywords:** Type I interferons, auto‐antibodies, HIV infection, opportunistic infections

## Abstract

**Background:**

Auto‐antibodies neutralizing the activity of type I interferons have been recently described in patients infected by SARS‐CoV‐2. They can be present even before the onset of the infection. Since type I interferons exert a dichotomous role in the pathogenesis of acute versus chronic HIV infection and auto‐antibodies are often found in untreated and anti‐retroviral treated HIV^+^ patients, we investigated whether auto‐antibodies anti‐type I interferons are present at high prevalence in those HIV^+^ patients with concomitant opportunistic infections (OIs).

**Methods:**

The analysis of auto‐antibodies against two types of type I interferons (IFN‐α2 and IFN‐ω) was performed using the ELISA test in 60 patients chronically infected by HIV who showed concomitant infections caused by mycobacterium tuberculosis or nontuberculosis mycobacterium or with active cytomegalovirus infections. Results were compared with those of 283 SARS‐CoV‐2 swab positive patients showing mild to severe pneumonia. A chi‐square (*χ* ^2^) test or the Wilcoxon–Mann–Whitney test were used to compare the HIV^+^ patient categorical or continuous variables, respectively.

**Results:**

A high prevalence of auto‐antibodies to type I interferons was found in middle‐aged HIV‐infected patients with concomitant OIs (11.6% vs. 5.3% in COVID‐19 subjects; *p* < .05). No statistically differences were found for viro/immunological characteristics (CD4 and CD8 cell counts and viral load) between patients with and without type I interferons auto‐antibodies.

**Conclusions:**

This study, which is the first searching auto‐antibodies against type I interferons in HIV‐infected patients, demonstrated that their prevalence was higher than that expected by the age of these patients. Furthermore, it indicated that these auto‐antibodies are nonspecifically increased in critical SARS‐CoV‐2 infection but can be found also in other infections.

## 1 INTRODUCTION

The clinical consequences of infections, either bacterial or viral, can vary among individuals and range between silent infection to life‐threatening conditions, demonstrating that pathogens are necessary but not sufficient to the disease development.^1^

The occurrence of primary or secondary immune‐deficiencies could foster the onset of multiple infections, so called “opportunistic infections” (OIs), and studies of severely immunocompromised HIV‐infected patients have shed light on infections that are extremely rare in immunocompetent individuals. However, the type and severity of natural or OIs could depend on the gravity of the immune dysfunction; therefore, genetic and immune determinants involved on the outcomes of infections are currently focus of research.^2^ For instance, at least 1% of cases of critical COVID‐19 pneumonia are associated with inborn errors of type I interferons (IFN‐I) immunity account for, while at least 15% of them are due to the occurrence of auto‐antibodies (Abs) neutralizing some IFN‐I, which are present even before the onset of the infection.^3^ These auto‐Abs can recognize the different IFN‐α subtypes and/or IFN‐ω, more rarely IFN‐β and even less IFN‐ξ and IFN‐k.^3^, ^4^ In the general population, they have been detected in blood samples of 0.5% of individuals aged 20–60 years, but as already described for various other auto‐Abs since the 1960s, their frequency increases proportionally with age reaching 4% in subjects over 70 years old and 7% in those aged 80–85 years.^3^, ^5^

IFN‐I pathway mediate numerous immune interactions during viral infections. It exerts a dichotomous role in the pathogenesis of acute vs chronic HIV infection^6^: initially, it blocks HIV replication delaying the disease progression, but chronic long lasting signaling is associated with altered immune activation, which exacerbates clinical conditions. It also plays a detrimental function on the outcomes of tuberculosis, by delaying the Th1 cell specific response to mycobacterium tuberculosis (MTB) in the lungs.^7^, ^8^ Cytomegalovirus (CMV) infection usually affects the salivary glands, kidneys, and other organs and usually is almost asymptomatic. However, viral replication can occur in immunocompromised patients and it may contribute to the damage to organs and their functions. In immunocompetent subjects, IFN‐I is involved in the lifelong control of CMV infection.^9^

Few studies have investigated whether other pathogens apart SARS‐CoV‐2 may take advantage of the presence of auto‐Abs recognizing IFN‐I (IFN‐I auto‐Abs). The aim of this retrospective exploratory cross‐sectional study is to study the prevalence of IFN‐I auto‐Abs in a cohort of HIV‐infected patients with MTB or nontuberculosis mycobacterium (NTB) disease or active CMV infections.

## 2 MATERIALS AND METHODS

### 2.1 Study population

In this retrospective cross‐sectional pilot study, we included HIV‐infected patients enrolled in the MASTER cohort,^10^ followed in our center (Department of Infectious and Tropical Diseases, University of Brescia, Italy), who satisfy the following criteria: (i) aged >18 years old; (ii) MTB or NTB diseases or CMV DNA positive in plasma; (iii) availability of frozen samples taken during these OIs; (iv) accessibility to demographics, clinical (including other OIs) and viro/immunological data.

Plasma CMV DNA was detected by using Abbott RealTime CMV kit with a sensitivity of 31.2 IU/mL (20 copies/mL). CMV disease was defined by histological evidence of inflammation and by the presence of CMV inclusion bodies confirmed by means of immunohistochemical staining.

MTB or NTB disease defined as presence of MTB or NTB in clinical samples of patients determined by polymerase chain reaction or culture.

Because samples have been collected before 2019 it is assumed that they were all SARS‐CoV‐2 negative.

We used samples of 283 HIV‐negative patients referred at the emergency room of the ASST Spedali Civili of Brescia with a tested swab positive for SARS‐CoV‐2 by real‐time polymerase chain reaction and showing mild to severe pneumonia^11^ as comparison group. Samples of patients that developed critical pneumonia were excluded.

### 2.2 Detection of IFN‐I auto‐Abs

Analysis of auto‐Abs against IFN‐α2 and IFN‐ω were performed using the ELISA method described in the Bastard et al.,^3^, ^4^ with few modifications. High protein‐binding capacity 96 well ELISA plates (Sarstedt ELISA‐plate, ref: 82.1581.200) were coated overnight at 4°C with 1 µg/mL of recombinant human IFN‐α2 (Myltenic Biotec, ref: 130‐093‐874) or IFN‐ω (Merck Life Science™, ref: SRT3061). Plates were washed three times with phosphate buttered saline (PBS) 1X, pH 7.4 (Merck Life Science, ref: P‐3813) and then incubated with PBS supplemented with 5% nonfat milk powder (Merck Life Science, ref: P‐4739) for 1 h at room temperature in agitation. Plates were then washed with PBS/0.005% Tween, and incubated with samples of patients or controls diluted 1:50 in PBS/0.005% Tween for 2 h at room temperature. Each sample was tested once in duplicate. Plates were thoroughly washed. Horse–conjugated Fc‐specific IgG fractions from polyclonal goat antiserum against human IgG (Nordic Immunological Laboratories, ref: GAHu/Ig (Fc)/PO) was added to a final concentration of 1 μg/mL. Plates were incubated for 1 h at room temperature in the dark and washed. Then, 3,3′,5,5′‐tetramethylbenzidine (LGC Clinical Diagnostics, ref: 5120‐007) substrate was added for 3 min. The reaction was stopped by adding 0.18 M sulfuric acid and optical density at 450 nm was measured. In each plate serum samples of positive controls, obtained from autoimmune polyglandular syndrome type 1 (APS‐1) patients (kindly provided by M.S. Lionakis, NIAID, NIH) and negative samples were consistently included.

All test values equal or greater than a cutoff of ≥0.7 O.D. are considered positive.

### 2.3 Statistical analysis

The mean values with standard deviation (SD) and median values were used to describe numerical variables, while the counts and percentages were employed for qualitative variables. A chi‐square (*χ* ^2^) test or the Wilcoxon–Mann–Whitney test were used to compare the groups for categorical or continuous variables, respectively.

### 2.4 Compliance with guidelines statement

This study has been performed in accordance with the relevant guidelines and regulations of the Declaration of Helsinki and Best practice guidelines.

### 2.5 Ethics approval and consent to participate

The present study was approved by Ethics Committee of Brescia (protocols NP 4000 and NP 3061), in accordance with current regulations (Legislative Decree no. 211 of June 24, 2003 and subsequent additions and authorizations), conducted in full respect of human dignity and fundamental rights, as dictated by the “Declaration of Helsinki” by the standards of “Good Clinical Practice” issued by the European Community (as implemented by the Italian Government and in accordance with the Guidelines issued by the same bodies), in implementation of what is also provided for by the Council of Europe Convention for the Protection of Human Rights and Dignity of the Human Beings in the application of biology and medicine made in Oviedo on April 4, 1997.

Patient data have been made anonymous (alpha‐numeric code) in observance of the rights provided for by privacy legislation (Legislative Decree no. 196/2003, Art. 7). Since this study was retrospective and nonpharmacological, informed consent has not been provided because, in Italy, ethical clearance for these studies is not needed (Italian Guidelines for classification and conduction of observational studies, established by the Italian Drug Agency‐ “Agenzia Italiana del Farmaco—AIFA,” on March 20, 2008). In addition, we used the general authorization of the Italian Guarantor for the use of anonymized demographical and clinical data.

## 3 RESULTS

Auto‐Abs against IFN‐α and IFN‐ω subtypes tested in 60 HIV‐infected patients with a mean age of 47 years (±10.29): 45 were male patients (75%; mean age: 47 years ±10.09), and 15 female patients (25%; mean age ± SD: 46.67 ± 11.22 years). We included 50 patients with positive plasma CMV DNA (83%); 30 of them had disseminated CMV (CMV diseases). MTB disease was present in nine (15%) and NTB disease in 10 (16.7%) patients (Table 1).

**Table 1:** Viro/immunological characteristics of HIV‐infected patients with (+) and without (−) IFN‐I auto‐Abs.

	IFN‐I auto‐Abs+ *N* = 7	IFN‐I auto‐Abs− *N* = 53	Total *N* = 60
Male, *n* (%)	7 (100)	40 (75.4)	45 (75)
Age, mean (±SD)	40.9 (**±**10.3)	52.9 (**±**9.3)	52 (**±**10.2)
CMV DNA positive, *n* (%)	3 (42.8)	47 (88.6)	50 (83.3)
MTB disease *n* (%)	2 (28.5)	7 (13.2)	9 (15)
NTB disease, *n* (%)	2 (28.5)	8 (15)	10 (16.7)
CD8+ count (cells/μL), mean (±SD)	820 (**±**512)	877 (**±**445)	871 (**±**627)
CD4+ count (cells/μL), mean (±SD)	262 (**±**134)	241 (**±**300)	243 (**±**284)
CD4+ at HIV diagnosis (cells/μL), mean (±SD)	123 (**±**94)	118 (**±**172)	118 (**±**163)
CD4+ Nadir (cells/μL), mean (±SD)	82 (**±**79)	83 (**±**104)	83 (**±**100)
HIV‐RNA <50 cp/mL, number of patients (%)	1 (14.2%)	24 (45.2%)	25 (41.6%)
HIV‐RNA at HIV diagnosis (copies/mL), mean (±SD)	2.3 × 10^6^ (**±**3.8 × 10^6^)	3.2 × 10^5^ (**±**4.8 × 10^5^)	5.8 × 10^5^ (**±**1.5 × 10^5^)
Other OIs^a^, *n* (%)	3 (42.8)	23 (43.3)	26 (43.3)

^a^ Opportunistic infections (OIs): pneumocistis carinii pneumonia, esophageal candidiasis, neurotoxoplasmosis.

Nineteen patients (19/60, 31.6%) had more than one active coinfections (MTB, NTB or the presence of plasmatic CMV DNA (Table 2).

**Table 2:** Coinfections.

	Mycobacterium tuberculosis	Nontuberculosis mycobacterium	Total
Cytomegalovirus (CMV) DNA	1	1	2
CMV disease	3	0	3
CMV negative	5	9	14
Total	9	10	19

Most of the patients had received antiretroviral therapy, but only 41% of them had plasmatic HIV RNA < 50 copies/mL at the moment of IFN‐I auto‐Abs testing. As comparison group we used samples from 283 patients with COVID‐19 with a mean age of 53.12 ± 12.14 years; 61.13% of them were males (173/283).

IFN‐I auto‐Abs were detected in 7 out 60 HIV‐infected patients (11.6%) and in 15 out 283 (5.3%) subjects with mild to severe COVID‐19 (*p* < .05). Six out seven HIV‐infected patients with IFN‐I auto‐Abs (85.7%) had auto‐Abs against both IFN‐α and IFN‐ω subtypes versus 9 out 15 (60%) of the patients with COVID‐19.

No statistically differences were found between viro/immunological characteristics of HIV‐infected patients with and without IFN‐I auto‐Abs (Table 1).

None of the 19 patients with more than one active coinfections was positive for IFN‐I auto‐Abs.

Notwithstanding the lack of statistically differences between groups, we observed that patients with IFN‐I auto‐Abs had higher HIV viral load at HIV diagnosis (2.3 × 10^6^ vs. 3.2 × 10^5^ copies/mL).

## 4 DISCUSSION

Here, we describe a high prevalence (11.6%) of IFN‐I auto‐Abs in a group of HIV‐infected middle‐aged patients with MTB, NTB diseases or plasma CMV DNA positive. No statistically differences in the characteristics between patients with and without auto‐Abs were found. Patients positive for auto‐Abs had higher HIV viral load at HIV diagnosis than patients who tested negative for IFN‐I auto‐Abs. The prevalence in the comparison cohort of COVID‐19 patients was significantly lower (5.3%).

The role of IFNs in the innate immune response is well known and several inborn errors of IFN‐I immunity have been described.^5^ For instance, the production of high levels of neutralizing IFN‐I auto‐Abs can be genetically driven and it has been described in several settings as young patients with APS‐1, carrying germline loss‐of‐function monoallelic or biallelic mutations in the autoimmune regulator (AIRE) gene, in subjects with hypomorphic mutations of RAG1 or RAG2 and combined immunodeficiency, in men with hemizygous mutations of FOXP3 and IPEX, and in women with heterozygous null mutations of X‐linked NEMO and incontinentia pigmenti.^5^ However, IFN‐I auto‐Abs can also be identified in patients without genetic abnormalities related to IFN‐I immunity, such as in patients with systemic lupus, thymoma or myasthenia gravis, or treated with IFN‐α or IFN‐β.^5^

In murine models, the absence of functional IFN‐I can cause CMV reactivation in latently infected endothelial cells^12^ and the experimentally depletion of IFN‐I by using neutralizing antibodies increased propensity of murine gammaherpesvirus‐68 (MHV‐68) reactivation.^13^

In humans, auto‐Abs neutralizing the activity of IFN‐I were detected four decades ago in a 77‐year‐old woman with disseminated zoster.^14^ Their presence was considered to be clinically silent in general population until the COVID‐19 pandemic onset, when it was correlated to severity of COVID‐19, mainly in males over 65 years old.^3^

Several other compelling studies have confirmed the link between IFN‐I auto‐Abs and COVID‐19 severity as reported by Arrestier et al.^15^ Currently, the presence of those auto‐Abs are waking up interest in the course of other infections. Very recent data reported that they are present in about 5% of cases of life‐threatening influenza pneumonia.^16^ A lower incidence of IFN‐I auto‐Abs (1.1%) was found in a cohort of critically ill patients with acute respiratory failure which, however, included both infectious (rhinovirus, influenza, parainfluenza, and seasonal coronavirus infections) and noninfectious etiologies.^17^

It remains largely unknown whether those auto‐Abs can be associated with severe nonrespiratory infections. IFN‐I auto‐Abs have also been detected in one‐third of subjects with severe adverse events following vaccination for yellow fever virus.^18^ In addition, very recently, Busnadiego et al.^19^ have described a potential link between the presence of IFN‐I auto‐Abs and possible reactivation of latent viruses infections, particularly herpesviruses (CMV, HSV‐1/2, or both) in patients with COVID‐19.

This is the first study searching IFN‐I auto‐Abs in HIV‐infected patients. We describe that their prevalence is higher than that expected by age. This could be due to the selection of patients included in this study who were those who also had the highest probability of having a more profound immunodeficiency, given the concomitant OIs.

The presence of auto‐Abs, in general, is not unexpected during untreated HIV infection and does not normalize once antiretroviral therapy is initiated.^20^ For instance, the presence of auto‐Abs against CD4 molecule seems to not have any clinical impact.^21^ Only the presence of anti‐cardiolipin IgG and total IgG have been described impact the clinical course of HIV infection.^22^

The significance of IFN‐I auto‐Abs found in HIV^+^ patients is difficult to be interpreted.

However, also the contribution of IFN‐I to both the control of HIV spread and the initiation of immunologic damage remains controversial despite over 30 years of research.^6^ The presence of IFN‐I auto‐Abs could be one of the factors that should be considered when interpreting the dichotomous role of IFN response in HIV immunopathogenesis, as well as the dynamic modulation of IFN production in acute versus chronic infection. This consideration can be applied for the OIs of our patients because IFN‐I has both detrimental and beneficial effects during human MTB and NTB infections and plays a role in the lifelong control of CMV infection.^23^ These contrasting effects could also be reflected on the role of IFN‐I auto‐Abs in our patients and could explain the lack of significant viro/immunological differences between patients with or without these auto‐Abs.

This study has some limitations. Being a pilot study, the sample size is relatively small, which might affect the generalizability of the findings, and its retrospective nature could have introduced biases and limitations in data collection. Therefore, future studies should consider a larger and more diverse sample size to strengthen the results and gain more comprehensive insights into the role of IFN‐I auto‐Abs in HIV‐infected patients with OIs. In addition, longitudinal studies with multiple time points and including patients from multiple centers and geographic regions would help establish temporal associations between IFN‐I auto‐Abs’ presence and the development of OIs in HIV patients and would enhance the external validity of the findings. Finally, additional mechanistic studies would elucidate the underlying mechanisms of IFN‐I auto‐Abs production and their impact on the immune response in the context of HIV infection and OIs.

From the methodological point of view, to enhance the robustness of the study, the presence of auto‐IFN‐I auto‐Abs should be assessed by alternative methods, such as Western blot analysis or other immunoassays. In addition, IFN‐I auto‐Abs should be tested in HIV‐infected patients without OIs and the IFN‐I neutralizing activity should be investigated in positive samples In conclusion, our data demonstrated that IFN‐I auto‐Abs are nonspecifically increased in patients infected by SARS‐CoV‐2 but can be found also in a subset of HIV‐infected patients with OIs.

In conclusion, our data demonstrated that IFN‐I auto‐Abs are nonspecifically increased in patients infected by SARS‐CoV‐2 but can be found also in a subset of HIV‐infected patients with OIs.

## AUTHOR CONTRIBUTIONS

**Luisa Imberti**: Conceptualization; data curation; investigation; writing—original draft; writing—review and editing. **Paola Magro**: Data curation; validation; writing—review and editing. **Alessandra Sottini**: Investigation; writing—review and editing. **Virginia Quaresima**: Investigation; writing—review and editing. **Francesco Castelli**: Supervision. **Eugenia Quiros‐Roldan**: Conceptualization; data curation; formal analysis; methodology; supervision; writing—original draft; writing—review and editing.

## CONFLICT OF INTEREST STATEMENT

The authors declare no conflict of interest.

## Data Availability

Data presented in the manuscript have not been published on the web. Data collected for this study are available for consultation on demand to the corresponding author.
